# Preparation and Characterization of Chitosan Poly(acrylic acid) Magnetic Microspheres

**DOI:** 10.3390/md8072212

**Published:** 2010-07-23

**Authors:** Liang Guo, Guang Liu, Ruo-Yu Hong, Hong-Zhong Li

**Affiliations:** 1 Department of radiology, The First Affiliated Hospital of Soochow University, Suzhou 215007, China; E-Mail: ilguoliang@sohu.com; 2 Key Laboratory of Organic Synthesis of Jiangsu Province, College of Chemistry, Chemical Engineering and Materials Science, Soochow University, SIP, Suzhou 215123, China; E-Mail: suppper@126.com; 3 State Key Laboratory of Multiphase Complex Systems, Institute of Process Engineering, Chinese Academy of Sciences, Beijing 100080, China; E-Mail: hzli@home.ipe.ac.cn

**Keywords:** magnetic microsphere, magnetic separation, nanoparticles, chitosan poly(acrylic acid)

## Abstract

Spherical microparticles, capable of responding to magnetic fields, were prepared by encapsulating dextran-coated Fe_3_O_4_ nanoparticles into chitosan poly(acrylic acid) (PAA) microspheres template. The obtained magnetic microspheres were characterized by transmission electron microscopy (TEM), Fourier transform infrared spectroscopy (FT-IR), scanning electron microscopy (SEM), X-ray powder diffraction (XRD), and thermogravimetry (TG). The results showed that the microspheres were formed and demonstrated magnetic behavior in an applied magnetic field. In addition, magnetite particles were well encapsulated and the composite particles have high magnetite content, which was more than 40%.

## 1. Introduction

Magnetic microspheres consisting of one or more magnetic nanoparticles have attracted a great deal of attention for their interesting chemical and physical properties. Magnetic microspheres find widespread and diverse application in many fields, such as environment remediation and therapeutic [1–3] and diagnostic biomedical applications [4–6]. In recent years, the research has been focused on synthesizing monodispersed, nanoscale spheres for high sensitivity and efficiency [7], achieving high saturation magnetization for fast and sensitive magnetic signal control, manipulation, and detection [7,8] and providing hydrophilic surfaces with various functional groups for covalent coupling to antigens, antibodies, enzymes, or DNA/RNA hybridization [9].

The magnetic microspheres are typically synthesized by various approaches such as the coprecipitation of ferrous and ferric salts under alkaline conditions in the presence of either polymers [10–12], heterogeneous polymerization methods, including suspension [13], dispersion polymerization [14], emulsion polymerization [15], miniemulsion polymerization [16,17], emulsifier-free emulsion polymerization [18], dispersion polymerization [19], suspension polymerization [20,21] and microemulsion polymerization [22]. Through emulsion copolymerization, it is possible to introduce suitable functional groups on the surface of polymer microspheres.

Chitosan and its derivatives have been widely used in many biomedical fields for their many significant biological and chemical properties (reactive groups such as -OH and -NH_2_). As a special functional material, the preparation of magnetic chitosan microsphere has been attracting the researchers for many years [23,24]. However, the chitosan is not suitable to bind directly onto Fe_3_O_4_ nanoparticles for that the resultant magnetic composites tend to aggregate for polymer cross-linking and physisorption. Many researchers are looking for suitable methods to modify chitosan and bind onto Fe_3_O_4_ particles. Chang *et al.* prepared carboxymethyl chitosan-Fe_3_O_4_ particles via carbodiimide activation [25,26]. The final polymer magnetic microspheres had a high magnetic content, and showed unique pH-dependent behaviors on the size and zeta potential.

The present work is aimed at the synthesis of new chitosan-poly(acrylic acid) (CS-PAA) magnetic microspheres and characterization of the magnetic properties and microstructure. We have prepared water-dispersible superparamagnetic Fe_3_O_4_ magnetic nanoparticles with 20–30 nm by the precipitation method in aqueous solution of dextran [27–29]. The chitosan-PAA microspheres were then prepared by polymerizing acrylic acid into chitosan template in aqueous solution. Finally, the Fe_3_O_4_ nanoparticles were well-encapsulated in chitosan-PAA microspheres by cross-linking with glutaraldehyde. The size, structure and magnetic properties of the resultant magnetic nanoparticles were characterized by X-ray diffraction (XRD), Fourier transform infrared spectroscopy (FT-IR), thermogravimetric analysis (TGA) and transmission electron microscopy (TEM).

## 2. Experimental

### 2.1. Materials

Ferric chloride (FeCl_3_·6H_2_O), ferrous sulfate (FeSO_4_·7H_2_O) and aqueous ammonia were all of analytic grade. Chitosan with the deacetylation degree (DD) of 90% and the molecular weight (Mw) of 200 kD was purchased from Kabo Biochemical Company (Shanghai, China). Dextran T20 (Mw ≈ 20,000) and glutaraldehyde (GA, 25% aqueous solution) were purchased from the SCRC (Sinopharm Chemical Reagent Co., Ltd.). Acrylic acid (Shanghai Chemical Reagents Company) (Shanghai, China) was distilled under reduced pressure in nitrogen atmosphere. Potassium persulfate (K_2_S_2_O_8_) was recrystallized from deionized water. All other reagents were of analytical grade and used without further purification.

### 2.2. Preparation of dextran magnetic fluid

The synthesis of dextran-MF was based on our previous study. In typical synthesis, a mixture of dextran and iron (III) chloride hexahydrate dissolved in 30 mL of deionized water was put into a three-neck flask equipped with a mechanical stirrer, and 0.5 mL hydrazine hydrate was added into the flask. After the mixture was stirred and mixed completely, some sulfate heptahydrate was added. After 15 min, sulfate heptahydrate was dissolved completely, and some ammonia solution was quickly dropped into the mixture with vigorous stirring under argon protection, followed by slowly dropping additional ammonia solution until the pH of the solution reached 10. Soon afterwards the solution was stirred for an additional 3 h in argon atmosphere at the temperature of 60 °C. The black suspension was cooled and purged by centrifugation at a speed of 7000 rpm for 20 min to separate large particles from the suspension, then the liquid at the top of the separation tube was taken out. Excess ammonia, hydrazine and its leftovers, iron cation and dextran macromolecules were removed by dialysis using a membrane bag with a 14,000 cut-off molecular weight for 24 h. The deionized water for dialysis was replaced every 1 h.

### 2.3. Synthesis of chitosan-PAA nanospheres by polymerization

The chitosan-PAA nanospheres were synthesized according to the method described as follows. Briefly, the chitosan-PAA nanospheres were obtained by polymerization of acrylic acid (AA) in chitosan solution. Chitosan was dissolved in 50 mL acrylic acid solution at the ratio of 1:1 ([aminoglucoside units]:[AA]) under magnetic stirring. The amount of AA was maintained constantly at 3 mmol in all experiments. Until the solution became clear, 0.1 mmol of K_2_S_2_O_8_ was added to the solution with continued stirring. The pH of the suspension was maintained at about 4.0. Then, the polymerization was carried out at 80 °C under a nitrogen stream and magnetic stirring. When the opalescent suspension appeared, the reaction system was cooled, and the opalescent suspension was filtered with paper filter to remove any polymer aggregates. Finally, the residual monomers were removed by dialysis in a buffer solution of pH = 4.5 for 24 h using a dialysis membrane bag with a molecular weight cut-off of 10 kDa. Replacing deionized water every 1 h.

### 2.4. Preparation of Fe_3_O_4_/chitosan-PAA polymer magnetic microspheres

Five mL of dextran-MFs was added to 20 mL of suspension solution of chitosan-PAA nanospheres. Then, 0.2 mL of 25% GA aqueous solution was added to this reaction system to cross-link chitosan, and the cross-linking reaction was allowed to proceed for 2 h. Finally, the resulting product was filtered to remove any possible aggregation. The unloaded magnetite nanoparticles were separated by three or more consecutive cycles whereby the suspension was ultracentrifuged (Ultra Pro 80, 8000 r/min, 277 K), the supernatant was discarded, and the wet solid was resuspended in water.

### 2.5. Characterization

Crystallographic studies of the pure Fe_3_O_4_, Fe_3_O_4_/chitosan-PAA microspheres were performed on an X-ray diffractometer (D/Max-IIIC, Japan) using Cu-kα radiation (λ = 1.5406 Å). Distances between peaks were compared to the JCDPS 5-0664 of International Center for Diffraction Data to determine crystalline structures.

Fourier transform infrared spectroscopy (FT-IR) analysis of the samples was taken on a Nicolet Avatar 360 Fourier transform infrared spectroscopy (FT-IR). Fourier transform infrared (FT-IR) spectra were obtained using KBr method.

The morphologies of dextran-coated Fe_3_O_4_ nanoparticles, chitosan-PAA nanospheres and Fe_3_O_4_/chitosan-PAA microspheres were observed by a Hitachi H-600-II transmission electron microscope (TEM) with an acceleration voltage of 200 kV. In this case, a drop of the dilute sample was deposited on a copper grid covered with a formvar-carbon membrane.

The magnetic properties of Fe_3_O_4_/chitosan-PAA microspheres synthesized by chitosan-PAA template were measured with a BHV-55 vibrating sample magnetometer (VSM).

Perkin-Elmer TGA-7 was employed to perform the thermo-gravimetric analysis (TGA). Dried sample (1–5 mg) was placed in the TGA furnace and the measurements were carried out under nitrogen with a heating rate of 20 °C/min from 50 °C to 900 °C.

## 3. Results and Discussion

### 3.1. X-ray diffraction (XRD) analysis

**Figure 1:**
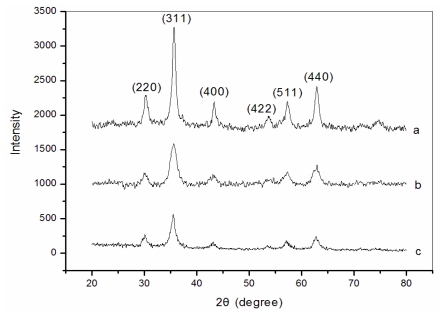
X-ray diffraction (XRD) spectrum: (**a**) naked Fe_3_O_4_; (**b**) dextran-coated Fe_3_O_4_; (**c**) Fe_3_O_4_/chitosan-PAA magnetic mirospheres.

Figure 1 shows the XRD patterns for naked (Figure 1(a)), dextran-coated (Figure 1(b)) and Fe_3_O_4_/chitosan-PAA (Figure 1(c)) nanoparticles, respectively. All the diffraction peaks are consistent with the seven diffraction peaks at (2 2 0), (3 1 1), (4 0 0), (4 2 2), (5 1 1), (4 4 0) and (5 5 3) by comparison with Joint Committee on Powder Diffraction Standards (JCPDS card, file No. 79-0418), which are indexed to the cubic spinel phase of Fe_3_O_4_. It reveals that the resultant nanoparticles are Fe_3_O_4_. The particle sizes can be quantitatively evaluated from the XRD data using the Debye-Scherrer equation, which gives a relationship between peak broadening in XRD and particle size.

(1) D=kλ/(β·cosθ)

where, *k* is Sherrer constant (0.89), λ the X-ray wavelength (0.15406 nm), β the peak width of half-maximum, and θ is the Bragg diffraction angle. The crystallite size of the Fe_3_O_4_/chitosan-PAA microspheres obtained from this equation was found to be about 100 nm. The peak positions of Fe_3_O_4_ nanoparticles are unchanged, which illustrated that the binding process did not result in the phase change of Fe_3_O_4_. The peak intensity of Fe_3_O_4_/chitosan-PAA composite particles is lower than that of dextran-coated Fe_3_O_4_ nanoparticles and naked Fe_3_O_4_ may due to that the Fe_3_O_4_ nanoparticles are incorporated into CA-PAA microspheres.

### 3.2. Fourier transform infrared spectroscopy (FT-IR) analysis

**Figure 2:**
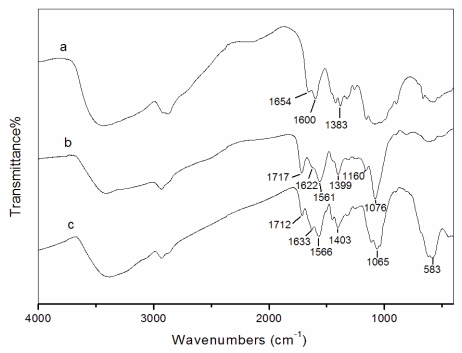
FT-IR spectra: (**a**) chitosan; (**b**) chitosan-PAA polymer microspheres; (**c**) Fe_3_O_4_/chitosan-PAA magnetic microspheres.

Fourier transform infrared spectroscopy (FT-IR) spectra of chitosan, chitosan-PAA polymer microspheres and Fe_3_O_4_/chitosan-PAA magnetic microspheres are shown in Figure 2. For the IR spectrum of chitosan (Figure 2(a)), the characteristic absorption bands appeared at 1654 cm^−1^ (amide I), 1600 cm^−1^ (amide II) and 1383 cm^−1^ (amide III). The absorption bands of the carboxyl groups of PAA and the -NH_2_ absorption of chitosan can be observed respectively at 1717 and 1622 cm^−1^ in the IR spectrum of chitosan-PAA nanoparticles (Figure 2(b)) and 1712 and 1633 cm^−1^ in that of Fe_3_O_4_/chitosan-PAA magnetic microspheres (Figure 2(c)). Furthermore, in Figure 2(c), the absorption peaks at 1566 and 1403 cm^−1^ could be assigned to asymmetric and symmetric stretching vibrations of COO^−^ anion groups. These indicate that the carboxylic groups of PAA are dissociated into COO^−^ groups which complex with protonated amino groups of chitosan through electrostatic interaction to form the polyelectrolyte complex during the polymerization procedure.

### 3.3. Transmission electron microscopy (TEM) and scanning electron microscopy (SEM)

**Figure 3:**
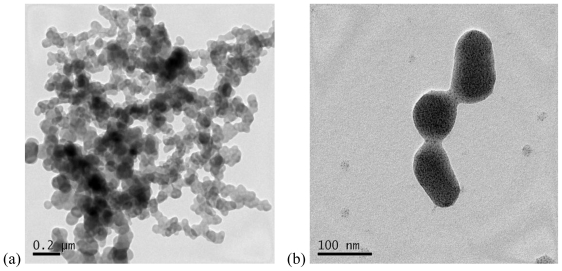
Transmission electron microscopy (TEM): (**a**) chitosan-PAA polymer microspheres; (**b**) Fe_3_O_4_/chitosan-PAA magnetic mirospheres.

**Figure 4:**
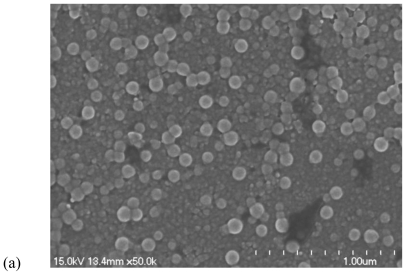

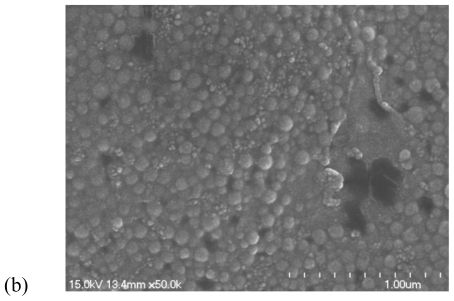
Scanning electron microscopy (SEM): (**a**) chitosan-PAA polymer microspheres; (**b**) Fe_3_O_4_/chitosan-PAA magnetic mirospheres.

The morphologies of chitosan-PAA microspheres and magnetic Fe_3_O_4_/chitosan-PAA composite microspheres were investigated by TEM (Figure 3) and SEM (Figure 4). Figure 3(a) shows the morphology of chitosan-PAA microspheres. It can be observed that the diameter of chitosan-PAA microspheres was about 100 nm. Figure 3(b) illustrates a TEM image of Fe_3_O_4_/chitosan-PAA composite microspheres, the average size of the sample is approximately 100 nm, and the results were reasonably consistent with those obtained from XRD. It can be seen from the TEM images that microspheres have excellent dispersibility and the composite particles are spherical in shape and the magnetite particles are encapsulated. The diameter of chitosan-PAA microspheres (Figure 4(a)) is consistent with those observed in Figure 4(b). In Figure 4(b), it can be seen clearly that the dextrancoated Fe_3_O_4_ was well encapsulated in the chitosan-PAA microspheres template.

### 3.4. Magnetic properties

**Figure 5:**
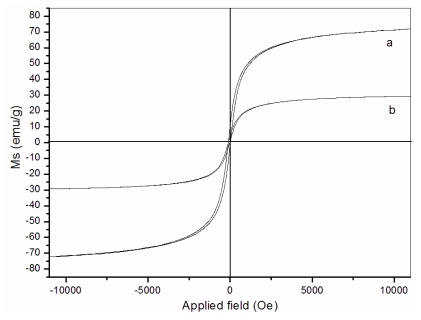
Magnetization curves obtained by vibrating sample magnetometer (VSM) at room temperature: (**a**) naked Fe_3_O_4_; (**b**) Fe_3_O_4_/chitosan-PAA magnetic microspheres.

The magnetization curves of the naked Fe_3_O_4_ particles (Figure 5(a)) and Fe_3_O_4_/chitosan-PAA composite microspheres (Figure 5(b)) recorded with VSM are illustrated in Figure 5. As shown in the figure, the magnetization of the samples would approach the saturation values when the applied magnetic field increases to 10,000 Oe. The saturation magnetization of naked Fe_3_O_4_ nanoparticles is 72.5 emu/g. For the Fe_3_O_4_/chitosan-PAA composite microspheres, the saturation magnetization is about 29.1 emu/g. The saturation magnetization of the nanoparticles was much less than that of bulk magnetite, which is 84 emu/g [30]. The lower value of the measured saturation magnetization is due to the smaller size of maghematite [31] and low saturation magnetization of Fe_3_O_4_/chitosan-PAA composite microspheres may be attributed to lager part in incorporation of Fe_3_O_4_ nanoparticles into chitosan-PAA spheres which added mass of the thick polymer layer on the magnetite nanoparticles. When the magnetic component size of the particles is smaller than critical size, the particles will exhibit superparamagnetism. A narrow hysteresis loop can be seen in Figure 5(a) and 5(b) respectively. There is a small remnant magnetization. It might be that some of the particles are magnetically blocked.

### 3.5. Thermogravimetric analysis (TG)

**Figure 6:**
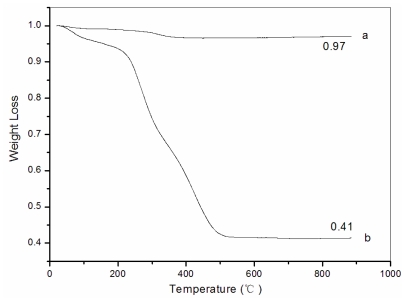
Weight loss curves: (**a**) naked Fe_3_O_4_; (**b**) Fe_3_O_4_/chitosan-PAA magnetic microspheres.

Thermogravimetric analysis (TG) was used to determine the weight percentage of Fe_3_O_4_ in the Fe_3_O_4_/chitosan-PAA composite microspheres. Fe_3_O_4_/chitosan-PAA composites were measured through the TGA runs in the condition of air atmosphere at the heating rate of 15 °C/min. Figure 6 shows the TGA curves of naked Fe_3_O_4_ nanoparticles and Fe_3_O_4_/chitosan-PAA magnetic microspheres. The TGA curve of naked Fe_3_O_4_ shows that the weight loss is about 3% for the whole temperature range. This might be due to the evaporation of absorbed or crystalline water. On the other hand, for Fe_3_O_4_/chitosan-PAA composites, below 200 °C the weight loss of all the nanocomposites is quite small (7%) because of the removal of absorbed physical and chemical water. Then the principle chains of chitosan-PAA begin to degrade at about 230 °C and the temperature of final decomposition is around 500 °C, the weight loss is significant (68%). There is no significant weight change from 500 to 900 °C, implying the presence of only iron oxide within the temperature range. Calculation results showed that the magnetic content of composite microspheres can be up to 41.3 wt%.

## 4. Conclusions

The Fe_3_O_4_/chitosan-PAA magnetic microspheres were successfully prepared by encapsulating dextran-coated Fe_3_O_4_ nanoparticles into chitosan-PAA microspheres template. Systematic investigations revealed that this synthetic approach is applicable for producing magnetic polymer microspheres with high magnetite contents. The saturation magnetization of composite nanoparticles could reach 29 emu/g and the nanoparticles showed superparamagnetism. The Fe_3_O_4_/chitosan-PAA magnetic microspheres have high stability of magnetization. The remarkable advantage of this system is that it is solely made of hydrophilic polymers: chitosan and poly(acrylic acid), which are non-toxic and biodegradable. Therefore, the composite spheres are favorable for their bioapplications.
